# Inhibition of HIV-1 Reverse Transcriptase-Catalyzed Synthesis by Intercalated DNA Benzo[a]Pyrene 7,8-Dihydrodiol-9,10-Epoxide Adducts

**DOI:** 10.1371/journal.pone.0072131

**Published:** 2013-09-19

**Authors:** Parvathi Chary, William A. Beard, Samuel H. Wilson, R. Stephen Lloyd

**Affiliations:** 1 Center for Research on Occupational and Environmental Toxicology, Department of Molecular and Medical Genetics, Oregon Health and Science University, Portland, Oregon, United States of America; 2 Laboratory of Structural Biology, National Institute of Environmental Health Sciences, National Institutes of Health, North Carolina, United States of America; University of Pittsburgh, United States of America

## Abstract

To aid in the characterization of the relationship of structure and function for human immunodeficiency virus type-1 reverse transcriptase (HIV-1 RT), this investigation utilized DNAs containing benzo[*a*]pyrene-7,8-dihydrodiol-9,10-epoxide (BPDE)-modified primers and templates as a probe of the architecture of this complex. BPDE lesions that differed in their stereochemistry around the C_10_ position were covalently linked to *N* ^6^-adenine and positioned in either the primer or template strand of a duplex template-primer. HIV-1 RT exhibited a stereoisomer-specific and strand-specific difference in replication when the BPDE-lesion was placed in the template versus the primer strand. When the C_10_ *R*-BPDE adduct was positioned in the primer strand in duplex DNA, 5 nucleotides from the 3΄ end of the primer terminus, HIV-1 RT could not fully replicate the template, producing truncated products; this block to further synthesis did not affect rates of dissociation or DNA binding affinity. Additionally, when the adducts were in the same relative position, but located in the template strand, similar truncated products were observed with both the C_10_ *R* and C_10_ *S* BPDE adducts. These data suggest that the presence of covalently-linked intercalative DNA adducts distant from the active site can lead to termination of DNA synthesis catalyzed by HIV-1 RT.

## Introduction

DNA polymerase interactions with their nucleic acid substrates have gained considerable interest in recent years with the determination of the crystal structures of several polymerase-DNA complexes, including but not limited to human immunodeficiency virus type-1 (HIV-1^1^) reverse transcriptase (RT) [1,2], A-family DNA polymerases (Klenow fragment of *Escherichia coli* DNA polymerase I [3], *Thermus aquaticus* DNA polymerase [4,5], *Bacillus stearothermophilus* DNA polymerase I [6], and T7 DNA polymerase [7]), and the X-family DNA polymerase β (pol β) [8,9,10]. Although the general shape of the polymerase domain is likened to a partially opened right hand with fingers, palm, and thumb subdomains, there is significant structural diversity in the composition of the residues that comprise these subdomains. The general features revealed by the structural characterization of these polymerase-DNA complexes have shown that polymerases interact with the duplex region of their nucleic acid substrates primarily through sugar-phosphate backbone and minor groove interactions. Further, it has been recognized that protein-DNA interactions occur primarily through sequence-specific DNA hydrogen bond donors and acceptors in the DNA major groove, while the minor groove offers very little hydrogen bond selectivity [11].

**Figure 1:**
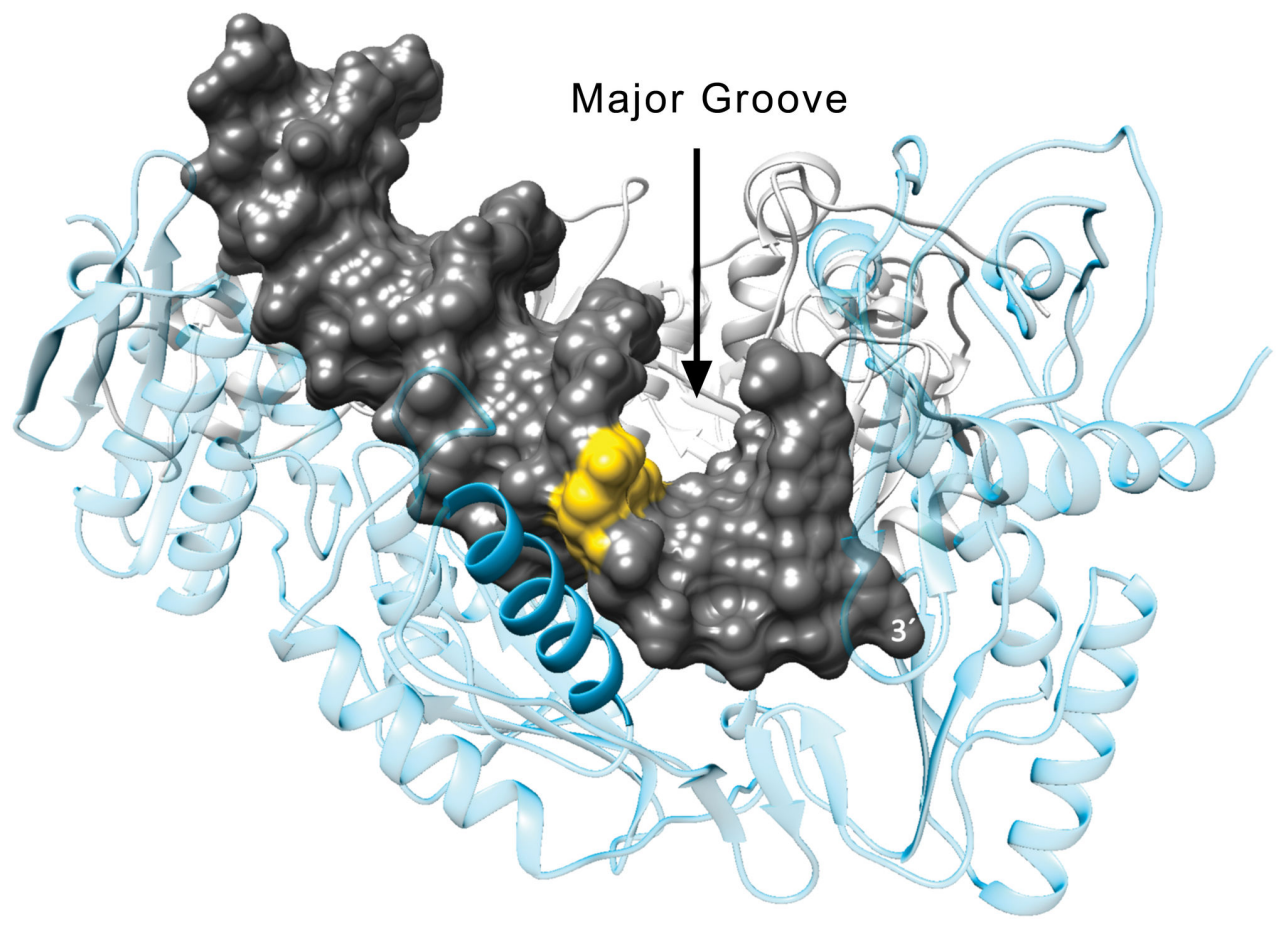
Structure of HIV-1 RT bound to DNA. The crystallographic structure of a T-P/HIV-1 RT complex is shown (PDB ID 3V4I). The heterodimeric HIV-1 RT is shown in a ribbon representation (p66, blue; p51, gray). The molecular surface of the DNA duplex is dark gray with the 5^th^ base pair upstream of the 3´-primer terminus (3´) colored yellow. The major groove is indicated and α-helix H (solid blue ribbon) is situated in the minor groove. The residues of the MGBT are on one side of the α-helix that face the DNA (residues not shown).

Additionally for the lentiviral reverse transcriptases, these polymerases have a series of amino acids in α-helix H that serve as a sensor of the DNA along its minor groove, the “Minor Groove Binding Track” (MGBT) [12]. The position of this helix places it in contact with newly synthesized duplex DNA ~2-6 nucleotides from the 3´ end of the primer strand (Figure 1). The structure of the DNA in this region is in the A-form near the polymerase active site and B-form near the RNase H domain, with a 40-45° bend at the junction. An α-helix in the thumb subdomain, helix H, interacts primarily with the primer strand in the region of the bend and contributes several key MGBT residues [12,13,14,15]. Previously, we have used site and stereospecific styrene oxide DNA adducts that were linked through either the *N* ^6^ exocyclic amino group of adenine or the *N* ^2^ exocyclic amino group of guanine, major and minor grooves, respectively as a probe of this MGBT interaction [16,17]. When the styrene oxide lesions were positioned in the minor groove, HIV-1 RT synthesis was terminated in the newly synthesized DNA, 4-7 nucleotides downstream of the lesions [16]. These data suggested that when α-helix H encountered the lesion, it became trapped with no further extension. When chemically identical adducts were positioned in the major groove, there was a compression of the corresponding minor groove and replication was blocked 1-3 nucleotides beyond the lesion [17]. These data are consistent with the interpretation that DNA adducts can indirectly terminate polymerization of HIV-1 RT by altering the flexibility of the DNA toward bending in the template-primer (T-P) stem and interfering with crucial enzyme-DNA contacts, especially those that involve α-helix H.

While these earlier studies provided insights into the molecular interactions between the α-helix H and the minor groove of the newly synthesized DNA, these analyses were limited by the fact that the styrene oxide adducts were completely localized in either the minor or major groove. However, other types of DNA adducts are not only able to covalently attach to an exocyclic amino group, but also intercalate into the hydrophobic DNA core. To further an understanding of the mechanisms by which HIV-1 RT-catalyzed synthesis can be blocked, we adopted an experimental design in which the *N* ^6^ position of adenine was chosen as the site for adduction by the polycyclic aromatic hydrocarbon, benzo[*a*]pyrene-7,8-dihydrodiol-9,10-epoxide (BPDE) that intercalates its aromatic rings into the helix [18,19,20,21]. Additionally, the design of the current experiments was expanded to position the lesion in either the template or the primer strand. This represents the first study to examine strand specific effects of DNA lesions on DNA synthesis.

## Materials and Methods

### Enzymatic construction and purification of BPDE-adducted and unadducted templates and primers

**Figure 2:**
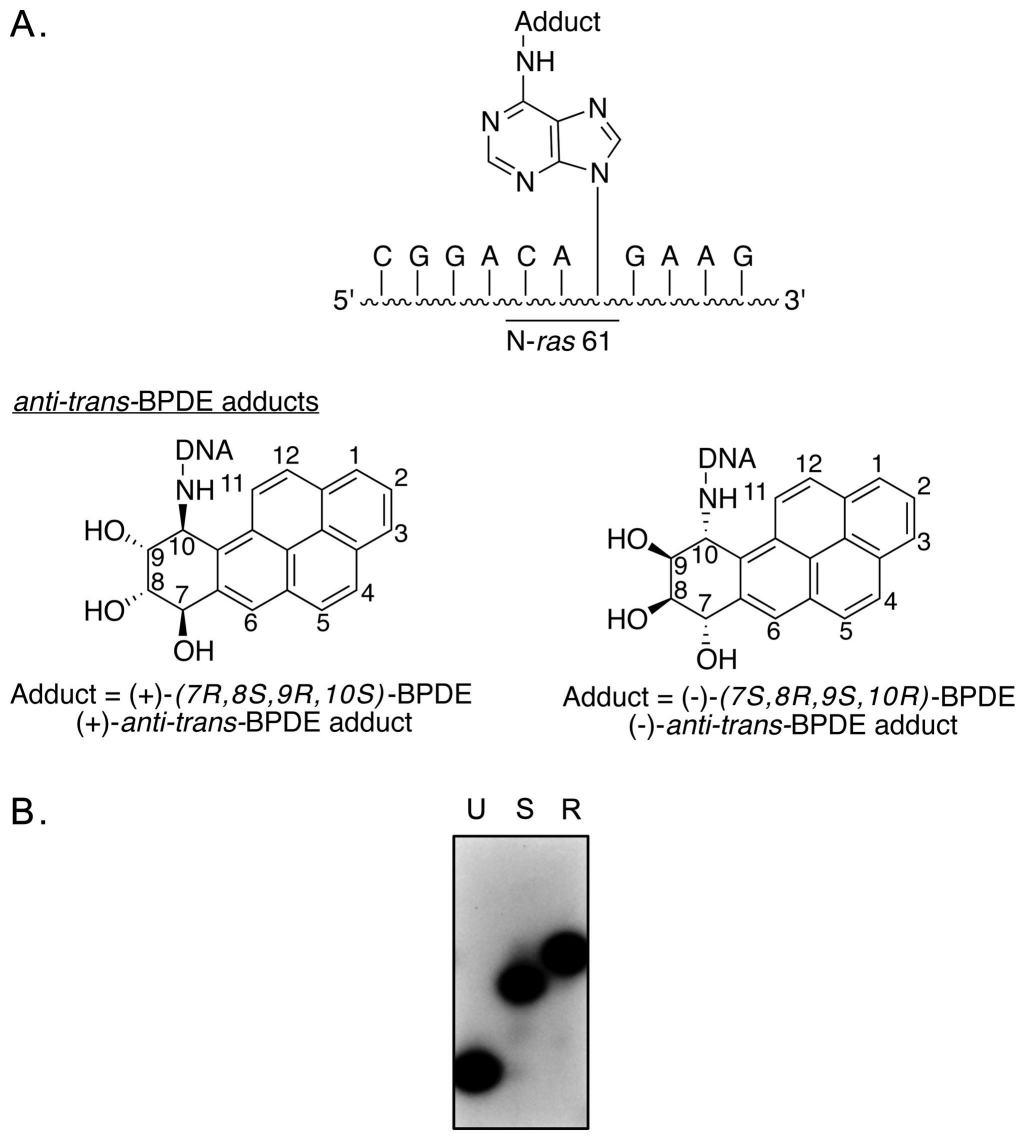
Structure and purity of stereoisomeric deoxyadenine *N* ^6^-BPDE adducts. (A) Structure of the (+) and (-) *anti-trans* BPDE that are covalently linked to adenine *N* ^6^ placed at the third position of N-ras codon 61 within an 11-mer oligodeoxynucleotide. (B) Autoradiogram of oligodeoxynucleotides that were ^32^P-labeled at the 5´-terminus and subjected to electrophoresis through 15% polyacrylamide gels. Lanes U, S and R represent unadducted, (*+*)*-anti-trans*- and *(-)-anti-trans*-BPDE-adducted templates, respectively.

**Figure 3:**
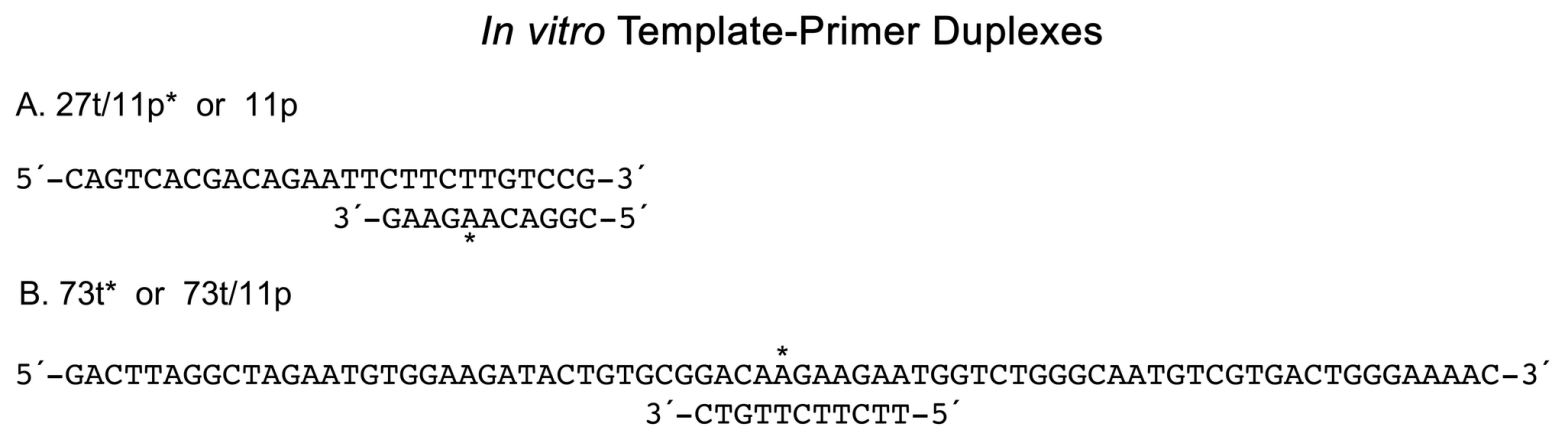
Nucleotide sequence and nomenclature of the templates and primers. Two sets of templates and primers were utilized in the primer extension reactions. In each case, the length of the template (t) and primer (p) is given. The position of the lesion is identified with a superscript in the adducted strand. A 27t/11p^*^ designation indicates that a 27-mer template has been annealed to an 11-mer primer that is adducted on the 5^th^ nucleotide from the 3΄-primer terminus.

Oligodeoxynucleotides (11-mers) containing the (+) or (-)-*anti-trans-*BPDE lesion were synthesized and purified as previously described (Fig. 2) [22,23]. The sequence context of the lesion is the third position of codon 61 of human N-ras gene. All unadducted control oligodeoxynucleotides were synthesized by Midland Research Inc. The 73-mer oligodeoxynucleotide strands (Fig. 3B) were constructed as follows. Using a 46-mer scaffolding DNA, the phosphorylated adducted or unadducted control 11-mers were ligated to both a 30-mer and 32-mer on the 5΄and 3΄ ends of the 11-mer, respectively. The 32-mer was also fully phosphorylated prior to ligation. The 25-mer oligodeoxynucleotide primers were created by ligation of a phosphorylated 14-mer to the adduct-containing or control 11-mer, respectively. All oligodeoxynucleotides were gel purified through 10% denaturing polyacrylamide gels.

As illustrated in Figure 3, two different constructs were made and utilized for primer extension reactions: a) the 27t/11p* represents a 11-mer adducted primer (5΄-CGGACA**A**GAAG-3΄) (p) annealed to a 27-mer template (t) in which the A with an asterisk (Figure 3) represents either C_10_ *S* or C_10_ *R* configuration of the BPDE-dA lesion located 5 nucleotides from the 3΄ terminus. The unadducted T-P is designated 27t/11p; b) the 73t*/11p represents an unadducted 11-mer primer annealed to a 73-mer adducted template containing either the C_10_ *S* or C_10_ *R* configuration of the BPDE-dA lesion (indicated by *). The adduct was located 37 nucleotides from the 3΄ end of the template strand within the duplex. The unadducted T-P is designated 73t/11p. Thus, in all sequence contexts, the adduct always remains in the same position.

### Enzymes and radiolabeled nucleotides

Sequenase version 2.0 (13 U/µL) was purchased from U.S. Biochemical (Cleveland, OH). Klenow fragment lacking proofreading activity (KF *exo* ^*-*^) was a gift from Dr. M.F. Goodman (University of Southern California, Los Angeles, CA). DNA polymerase β (pol β) [24] and HIV-1 RT [25] were purified as described previously. Ultrapure deoxynucleoside triphosphates were purchased from Pharmacia (Piscataway, NJ),

### Polymerase arrest assays

Oligodeoxynucleotide primers (11-, 20-, and 25-mers) were 5΄ end-labelled with (γ^32^P) ATP (6000 Ci/mmol, DuPont-New England Nuclear, Boston, MA) using T4 DNA kinase (New England BioLabs) and purified from excess ATP by column chromatography. Extension reactions with the 11-mer primers were performed at 18 °C for 30 min, whereas the reactions using longer primers were conducted at 37 °C for 30 min. The template (250 fmol) to primer (50 fmol) ratio was 5 in all cases. The primer to polymerase ratio was also 5 (10 fmol of polymerase). The reaction mixture contained 0.1 mg/mL bovine serum albumin, 1 mM dithiothreitol, 1.25 mM ATP, 300 µM dNTPs, 33 mM TrisOAc (pH 7.8), 66 mM KOAc, and 10 mM Mg(OAc)_2_. Extension reactions were terminated by the addition of 5 µL of stop buffer (95% (v/v) formamide, 20 mM EDTA, 0.05% (w/v) bromphenol blue, 0.05% (w/v) xylene cyanol) to the 10 µL reaction mixture. Reaction products were separated on sequencing gels (15% polyacrylamide and 8.3 M urea) by electrophoresis at 2000 V for 3 h and quantified by phosphorimager analysis.

### Primer-template enzyme binding assay

Solution conditions were similar to those described above for the polymerase arrest assay. HIV-1 RT (2.5, 5, or 10 fmol) was preincubated with adducted or unadducted 5´-^32^P-labeled T-P (i.e., 27t/11p*, 27t/11p; 73t*/11p, 73/11p) at 18 °C for 30 min in the absence of dNTPs. The template (250 fmol) to primer (50 fmol) ratio was 5 in all cases. After the initial incubation with the 27t/11p or 11p*, a competitor 33t/29p T-P was added in five-fold excess with dTTP, dCTP, and dGTP (330 µM each) and incubated at 18 °C. The competitor DNA binds free enzyme and enzyme that dissociates from the duplex used in the preincubation phase. DNA synthesis does not occur on the competitor T-P since the first two templating nucleotides are thymidines and dATP is omitted. The 29-mer primer of the competitor T-P was ^32^P-labeled to monitor binding and extension. Aliquots were taken at 0.5, 3, 10, 20, and 30 min and analyzed as described above. When the 73t or 73t*/11p was preincubated with HIV-1 RT, the competitor T-P was 27t/11p. In this situation, the only dNTP added was dATP along with the competitor DNA. The initial templating sequence of the 73t/11p is 3΄-GCG-5΄. The sequence of the 33-mer template and 29-mer primer were: 5΄-CGGACAAGAAGAATTCGTCGTGACTGGGAAAAC-3΄ and 5΄- GTTTTCCCAGTCACGACGAATTCTTCTTG-3΄, respectively.

## Results

### Primer extension analysis with unadducted (27t/11p) and C_10_ *S* or C_10_ *R* adducted 11-mer primers (27t/11p*)

Analyses of the co-crystal structures of HIV-1 RT with T-P DNA reveal that the enzyme makes a series of minor groove contacts in the newly synthesized duplex DNA. Additionally both major and minor groove lesions block the progression of HIV-1 RT that were located 1-3 and 4-7 nucleotides, respectively beyond the site of synthesis [16,17]. These data suggest that bulky DNA lesions can probe the interactions of the polymerase with newly synthesized DNA. However, the location of the previous adducts was limited to either residing in the major or minor groove and have not considered intercalation as an additional probe of the HIV-1 RT-DNA interaction.

**Figure 4:**
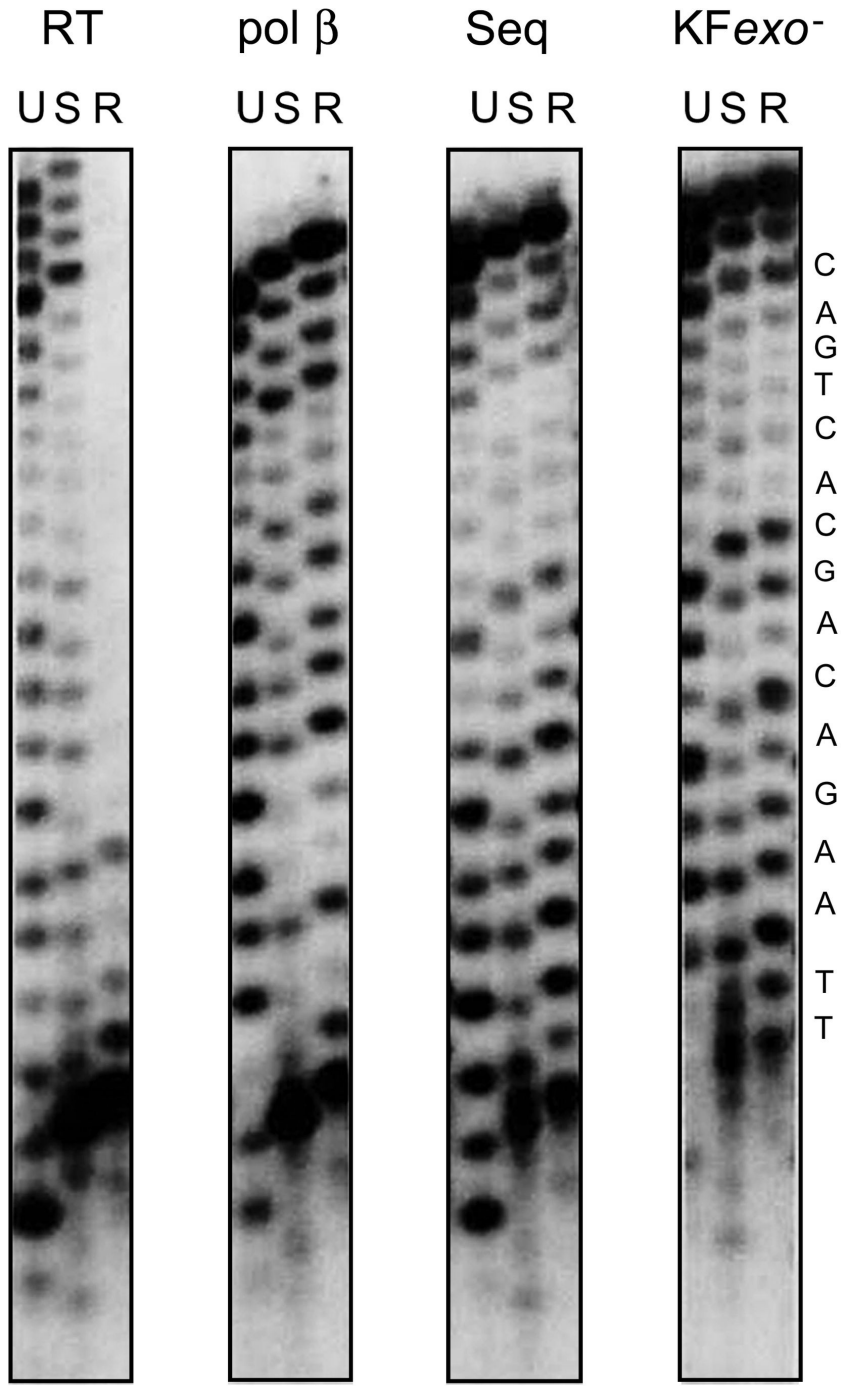
Primer extension analysis on unadducted and BPDE-adducted primers with alternate DNA polymerases. Polymerase extensions of 27t/11p^*^ or 11p were performed as described in MATERIALS and METHODS. In all cases, 10 fmol of enzyme was used. The primer to polymerase ratio was 5. U, S, and R represent unadducted, (+) and (-)*-anti-trans*-BPDE-adducted primers, respectively. The unadducted and adducted primers have different electrophoretic mobilities that result in products of different mobilities. Panels represent primer extension assays in the presence of the respective polymerase: HIV-1 RT(RT), DNA polymerase β (pol β), Sequenase (Seq), KF exo^-^ (KF exo^-^). The template sequence on the right coincides with full-length products that can be obtained by utilizing an 11-mer primer. The formation of truncated products is unique only to the *R*-adducted primer extended by HIV-1 RT. In some instances, non-templated blunt-end additions are observed (e.g., HIV-1 RT).

In order to investigate the replicative consequences of an intercalating DNA lesion, C_10_ *S*- or C_10_ *R*-BPDE adducts were positioned in the primer strand in the vicinity of the 45° DNA bend observed in the HIV-1 RT/DNA crystal structure [1]. The adducted and unadducted primers and full-length products exhibit differential electrophoretic mobilities due to the presence or absence of the BPDE adduct (Figure 4). Comparative analyses of unadducted primer extensions (27t/11p) with the BPDE-adducted (27t/11p*) revealed that HIV-1 RT was able to readily extend the unadducted (U) and C_10_ *S*-BPDE (S) adducted 11-mer primers to form longer and full-length products (Figure 4, RT, U and S, respectively). However in contrast, primer extension was severely restricted when HIV-1 RT utilized the C_10_ *R*-BPDE (R) adducted 11-mer primer (Figure 4, RT, R) with only a modest amount of single nucleotide extension and no replication detected beyond four incorporated nucleotides. These data demonstrate clear differences in the ability of HIV-1 RT to form productive polymerase complexes based on the BPDE stereochemistry within the primer strand. Additionally, these data are qualitatively similar to results obtained for HIV1-RT replication with templates containing *N* ^2^ G styrene oxide lesions [16].

In order to determine if this inhibition of extension using the C_10_ *R*-BPDE-containing primer was unique, other DNA polymerases were also examined in order to compare their replication patterns relative to HIV-1 RT. In contrast to the data generated for HIV-1 RT, pol β (Figure 4, pol β), Sequenase (Figure 4, Seq), and KF exo^-^ (Figure 4, KF exo^-^) catalyzed efficient primer extensions on both control and adduct-containing primers. Specifically, for these other polymerases, there were no significant differences in the utilization of primers containing *R* or *S* adduct stereochemistries. These data therefore demonstrate that ongoing DNA synthesis by HIV-1 RT is strongly and specifically inhibited when the primer strand contains the C_10_ *R*-BPDE intercalated major groove adduct 5 nucleotides upstream of the polymerase active site.

### Primer extension analyses with unadducted (73t/11p) and C_10_ *R* or C_10_ *S*-adducted 11-mer templates (73t^*^/11p)

**Figure 5:**
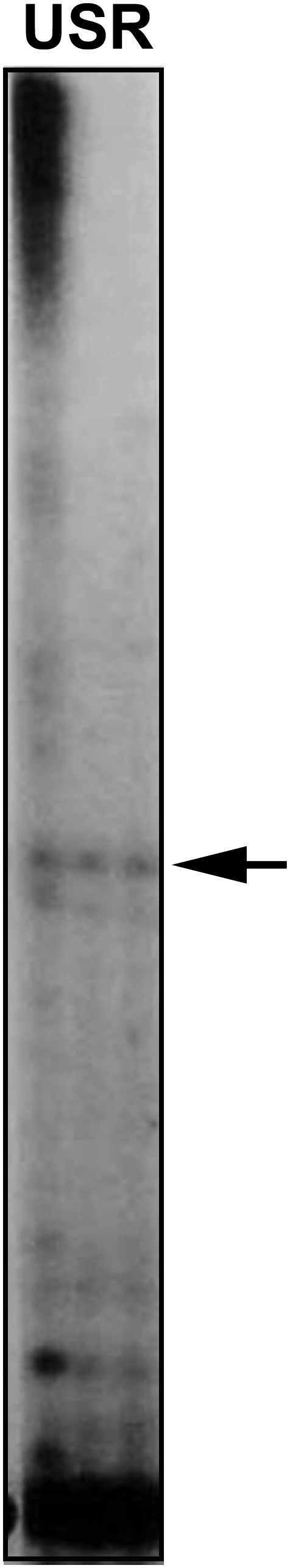
Primer binding and extension by HIV-1 RT on unadducted and C_10_ *R*- or C_10_ *S*-adducted templates. Primer extension assays using 11-mer primers on unadducted (73t) and adducted templates (73t^*^) were performed as described under MATERIALS and METHODS. U, S, and R represent unadducted, (+) and (-) *anti-trans-*BPDE adducted templates, respectively. The *R*- and *S*-adducted templates do not allow the formation of full-length products. The major site of termination after 11 incorporations is indicated.

In order to determine if the same DNA lesions located in the complementary strand would similarly inhibit HIV-1 RT primer extension, long oligodeoxynucleotides (73-mers) were constructed such that the adducted nucleotides were centrally located, with the primer strand annealed opposite the base modification. As shown in Figure 5, on the control primer extension reaction using the unadducted (U) T-P duplex, HIV-1 RT catalyzed efficient synthesis, resulting in a majority of DNA products being full-length, with a very minor termination site observed following incorporation of 11 additional nucleotides. In contrast, unadducted primers bound to templates containing C_10_ *R*- and C_10_ *S*-BPDE adducts were very poorly extended and the minor population of extended primers were terminated with a pattern similar to that observed for the unadducted template (Figure 5, lanes S and R). These data suggest that when either the C_10_ *R*- or C_10_ *S*-BPDE adducts was positioned within the portion of the newly synthesized DNA that interacts with α-helix H in RT, these templates cannot support replication.

### Binding affinity of HIV-1 RT on primer-template complexes containing BPDE adducts

**Figure 6:**
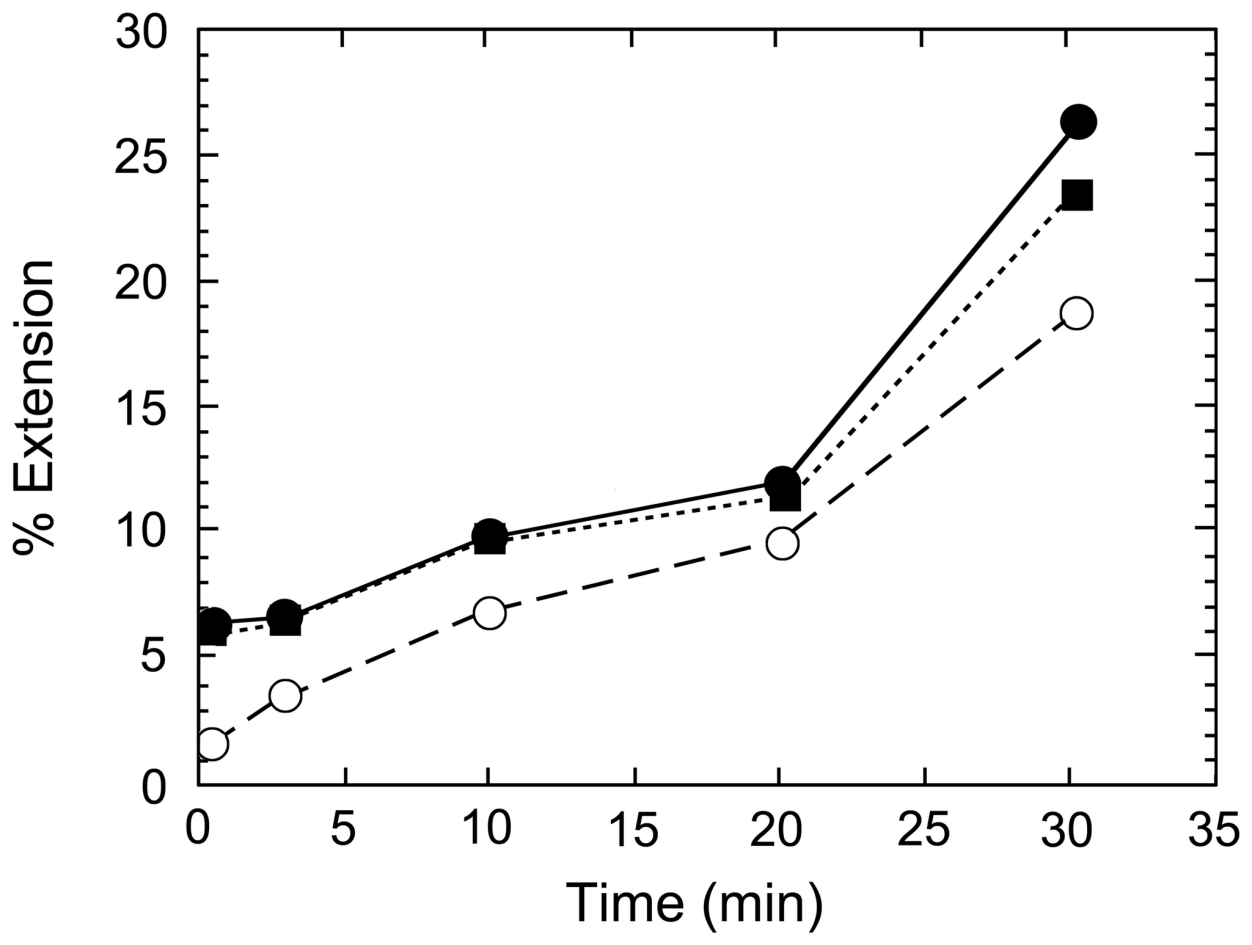
Relative dissociation rate constants from adducted or unadducted DNA. Kinetic analyses of the extension of labelled primer (33t/29t) when 2.5 fmol of HIV-1 RT was preincubated with unadducted (open circles), 27t/11p C_10_ *R* (closed circles)- or C_10_ *S* (closed squares)-adducted 11-mer primers (27t/11p^*^). U, S, and R represent unadducted, (+) and (-) *anti-trans-*BPDE adducted templates, respectively.

To determine whether the adducted 11-mer primer (Figure 4) influenced the apparent DNA binding affinity of HIV-1 RT, a competition binding/dissociation and polymerization assay was used to estimate the HIV-1 RT dissociation rate constant from unadducted and adducted primers (Figure 6). The dissociation rate constant is a sensitive monitor of overall DNA binding affinity, since the association rate constant, *k*_on_, is often diffusion-controlled [26]. HIV-1 RT was preincubated with an excess unlabeled unadducted T-P, containing either control unadducted or the C_10_ *R*- or C_10_ *S*-adducted t-p (27t/11p or 27t/11p^*^) in the absence of dNTPs to form a polymerase/DNA complex. To measure relative rates of dissociation, a different ^32^P-labelled T-P was added concomitantly with dTTP, dCTP, and dGTP. The experimental design severely restricted nucleotide incorporation on the original unadducted or adducted unlabeled complex, since the first two templating nucleotides were dT. Enzyme dissociations from each of the original complexes were monitored by elongation of the competitor DNA. Data shown in Figure 6 reveal a very similar rate of competitor primer extension, suggesting comparable dissociation/reassociation rates for the adducted and unadducted 11-mer primers.

## Discussion

Insights into the molecular interactions between two macromolecules such as polymerases and DNA can be obtained either by altering specific resides in the enzyme by site-directed mutagenesis or by modifying the T-P complex by covalently attaching stereospecific adducts on either the template or primer strands. Deoxyadenosine adducted at *N* ^6^ with BPDE has been employed previously in single-stranded templates to examine translesion bypass synthesis with a variety of DNA polymerases [27,28,29]. In many instances, DNA polymerases terminate synthesis one nucleotide prior to, opposite, or beyond the lesion. The exact site of termination was dependent on the identity of the polymerase, the absolute configuration at C_10_ (*R* versus *S*), and the local sequence context. As part of these previous studies, it was shown that HIV-1 RT generally terminated DNA synthesis one nucleotide prior to both the (-)-*anti-trans-*C_10_ *R* and (+)-*anti-trans*-C_10_ *S* BPDE adducts when DNA synthesis was limited to one polymerase encounter per T-P binding event. Under conditions that allowed multiple polymerase encounters, bypass DNA synthesis was observed with (+)-*anti-trans*-C_10_ *S* BPDE, but termination occurs one nucleotide 5΄ to the (-)-*anti-trans*-C_10_ *R* adduct. Blockage of DNA synthesis by the C_10_ *R* adduct could not be overcome by annealing a primer in which the 3΄- terminus was one nucleotide beyond the lesion [27]. These data demonstrate that under a variety of T-P conditions, the (-)-*anti-trans*-C_10_ *R* BPDE adduct was significantly more disruptive to polymerization than the corresponding (+)-*anti-trans*-C_10_ *S* BPDE adduct. Stereochemical, structural and thermodynamic analyses of these two adducts in the same CAA sequence context provided significant insights into understanding the origins of these differences [19]. This study extended prior NMR analyses of duplex DNAs containing these lesions [18,20,21], in which molecular dynamics simulations revealed that the C_10_ *R*-containing DNA was more stable by ~13 kcal/mol compared to the C_10_ *S*-containing DNA. This destabilization is at least partially driven by *syn-anti* conformational heterogeneity around the glycosyl bond, resulting in diminished base stacking, helix unwinding and poor Watson-Crick base pairing.

In the current study, to probe polymerase DNA-interactions in the duplex region of the T-P, two stereoisomers of BPDE were positioned 5 nucleotides upstream of the 3΄-OH of the primer strand or at a comparable position in the complementary template strand (Figure 3). Using these substrates, pol β, Sequenase, and KF exo^-^ were able to efficiently extend both the BPDE-adducted primers and unadducted primers that were annealed to a template containing the lesion. The crystal structures of these polymerases bound with DNA indicate that non-specific interactions occur with the DNA sugar-phosphate backbone and the minor groove [1,3,4,30]. These structures, as well as the high resolution structures of a pol I family polymerase bound to DNA [31], indicate that minor groove interactions can occur up to 5 base-pairs from the polymerase active site, and that the sugar-phosphate backbone interactions extend even further. The lack of a significant perturbation of primer extension by pol β, Sequenase, or KF exo^-^ for the C_10_ *R*- or C_10_ *S*-BPDE primers in which the adducted site was positioned 5 nucleotides into the duplex; suggest that this lesion does not significantly affect these polymerase sugar-phosphate backbone or minor groove interactions with these polymerases. Since a dramatic bend in the DNA duplex 4-5 nucleotides upstream of the polymerase active site is only observed in crystallographic structures of HIV-1 RT (1,2), this bend must alter local dynamic properties of the surrounding nucleotides that are sensitive to DNA lesions and modulate events at the polymerase active site. Our prior investigations corroborate these conclusions such that minor groove DNA adducts severely block ongoing replication as the lesion passes through this helix-induced bend [16]. Similarly, adducts in the major groove that compress the width of the minor groove also restrict HIV-1 RT transit through this bend. These inhibitory effects are not seen with other DNA polymerases that do not induce this bend.

In contrast, the present study focused on the influence of adducts placed either in the template or primer strands on HIV-1 RT catalyzed DNA polymerization. In contrast to the other polymerases examined, 11-mer primers containing the C_10_ *R*-BPDE adduct or templates containing either the C_10_ *R*- or C_10_ *S*-BPDE adduct were strongly inhibitory to primer extension. Importantly, nucleic acid binding, processivity, and frameshift fidelity are influenced by hydrophobic and hydrogen bonding interactions within the DNA minor groove and a group of amino acid residues in the thumb subdomain, referred to as the MGBT that are highly conserved among lentiviral reverse transcriptases [12,14,15].

Similar to that observed with the other DNA polymerases examined in this investigation, HIV-1 RT readily extended the C_10_ *S*-BPDE adducted 11-mer primer (Figure 4). In contrast, under the same conditions, the C_10_ *R*-BPDE adducted 11-mer primer was primarily extended by only a single nucleotide. As described above, these data are highly consistent with the findings by Yan et al [19],, who demonstrated major differences in the overall structures and stabilities of DNAs containing these adducts. The inability to extend the C_10_ *R*-adducted primer was shown to be due to a diminished rate of nucleotide incorporation and not due to an increased dissociation rate constant for the T-P complex. The unadducted and adducted primers or templates had similar DNA binding affinities as judged by their apparent dissociation rate constants (Figure 6).

Since the adduct can be perturbing in either the primer or template strands when it is positioned 5 base pairs from the polymerase active site, DNA lesions adducted in the major groove must be able to structurally alter the minor groove that is monitored by the MGBT of HIV-1 RT. The MGBT interacts primarily with the primer strand in the minor groove two to six nucleotides upstream of the polymerase active site and alterations of these interactions decrease DNA binding and polymerase processivity [12]. We have previously noted that DNA lesions adducted in the major groove influence RT termination in the vicinity of the MGBT [17]. The solution structure of the C_10_ *R* *N* ^6^-deoxyadenosine-BPDE adduct in duplex DNA indicates that the pyrenyl moiety is intercalated in the helix from the major groove on the 3΄ side of the modified adenine [18,20,21]. This results in distortion of the adjacent base pairs altering the position of the purine and pyrimidine hydrogen bond acceptors in the DNA minor groove (N3 and O2, respectively). In contrast to the premature termination observed with the C_10_ *R*-adduct, the C_10_ *S*-adduct positioned in the primer strand did not perturb important polymerase-DNA interactions. The low T_m_ of a duplex C_10_ *S* *N* ^6^-deoxyadenosine-BPDE opposite a complementary thymine base has precluded the determination of the solution structure of this stereoisomer [18,32]. However, the solution structure of the C_10_ *S*-deoxyadenosine adduct when positioned opposite a deoxyguanosine, indicates that the duplex exists in at least two conformers. The torsion angle of the glycosidic bond of the modified base adopts a *syn*-configuration in the major conformer [32] and an anti-configuration in the minor conformer [33]. The adduct was inserted into the helix on the 3΄-side of the modified adenine with both conformers [32,33]. The lack of an effect on HIV-1 RT termination suggests that the C_10_ *S*-BPDE adduct does not significantly impact the DNA minor groove when positioned opposite thymidine. Furthermore, as shown in Figure 5, although synthesis from primers bound to both unadducted and adducted 73-mers showed minor pause sites, the unadducted template could be extended to full-length products. In contrast, only very modest replication could be initiated off of *both* of the BPDE-adducted templates. Importantly, this indicates that the major groove adducted templating adenine signifiicantly alters DNA minor groove interactions with RT in the vicinity of the 40-45° bend in the duplex DNA upstream of the primer terminus and highlights the strand specific nature of the influence of the C_10_ *S*-BPDE adduct on RT replication. 
